# Summer Excursions

**Published:** 1871-04

**Authors:** 


### SUMMER EXCURSIONS.

Many are now forming plans for spending the summer. It is of secondary importance to decide where to go. The chief point is to go somewhere, north, south, east, or west. It is the change that does the good, — change of air, change of scene, change of food, of the mode of its preparation, change of associations, so as to have some new kind of excitement; such a change as will bring into requisition a different set of muscles, of mental faculties, of moral qualities, of social and domestic enjoyments; leaving at home, as far as possible, business cares, plans, schemes, labors, solicitudes.

There is some choice in localities; the hill country is better than the prairie; the mountain top, better than the valley.

The kind of occupation is important; it is the out-door life that does most of the good — the sunshine and the breeze. If a person goes to the country to eat, and sleep, and lounge about the house, but little benefit can be expected; but if nearly the whole time from after breakfast until sundown is spent in excursions on foot, on horse, in country wagons, — going every day to some point of interest, instructive and new, raising the spirits, stimulating inquiry, cultivating the better qualities of our nature, social, moral, and domestic, — then will benefit be derived; a marked and enduring benefit to mind, and heart, and body. Those who have any ailment of the throat or lungs should keep away from lake, and sea-shore, and prairie, because dangerous, raw, bleak, piercing, chilling winds follow the rains even of summer; besides, there is something in the sundown dampness of the sea-shore which is hurtful to weak lungs.

If persons are confined to the house, home is the best place for every sickness; there is no compensation under any hired or stranger's roof which can compensate for the quiet, and freedom, and roominess of one's own home; for the tender and sympathetic ministrations of friends and kindred. The risk is terrible, — the risk of dying among strangers. It is a beautiful parting prayer of one of the Eastern nations, "May you die among your kindred."

